# The clinical significance and function of miR-146 in the promotion of epidural fibrosis

**DOI:** 10.1590/1678-4685-GMB-2020-0447

**Published:** 2021-05-14

**Authors:** Yuan Fang, Xiaoli Hu, Shuzhen Liu, Yunwen Zou, Zhijie Wang, Yanchen Chu

**Affiliations:** 1 The Affiliated Hospital of Qingdao University, Department of Joint Surgery, Qingdao, Shandong, China.; 2 Women and Children’s Hospital of Linyi City, Department of Obstetrics and Gynecology, Linyi, Shandong, China.; 3 Medical Department of the Affiliated Hospital of Qingdao University, Qingdao, Shandong, China.; 4 The Affiliated Hospital of Qingdao University, Department of Spinal Surgery, Qingdao, Shandong, China.

**Keywords:** Epidural fibrosis, postoperative epidural scar formation, miR-146, fibroblasts proliferation, inflammatory response

## Abstract

Epidural fibrosis is the main cause of failed back surgery syndrome. To investigate the role of miR-146 in the diagnosis and development of epidural fibrosis. Lumbar disc tissues were collected from 72 lumbar disc herniation patients (45 developed epidural fibrosis and 27 did not). The expression of miR-146 in collected tissues and isolated epidural fibroblasts was detected by RT-qPCR. The relative levels of pro-inflammatory cytokines were analyzed by ELISA. The effect of miR-146 on the proliferation of fibroblasts was evaluated by MTT assay. miR-146 was significantly upregulated in epidural fibrosis patients compared with control patients. The expression of miR-146 was closely associated with the location, lower limb symptom and duration of disease of epidural fibrosis patients, and was positively correlated with the relative levels of pro-inflammatory cytokines. Moreover, miR-146 could discriminate epidural fibrosis patients from control patients. In isolated epidural fibroblasts, the overexpression of miR-146 dramatically enhanced its proliferation and the inflammatory response. miR-146 serves as a diagnostic biomarker for the early detection of epidural fibrosis. The upregulation of miR-146 enhanced the fibroblasts proliferation and inflammatory response in epidural fibrosis. This study provides a novel potential therapeutic target for epidural fibrosis.

## Introduction

Laminectomy is a routine surgical procedure in the treatment of lumbar disc herniation, lumbar spinal stenosis, and other spine diseases, which can relieve pain in the waist and lower extremities and promote the recovery of patients (Alkalay et al., 2003). Postoperative epidural scar formation, also called epidural fibrosis, is the body’s response to trauma after spinal surgery, especially in laminectomy (Manchikanti and Singh, 2002). The inflammatory response and hematoma formation after laminectomy are the main factors that result in the proliferation of fibroblasts, the accumulation of collagens, and the formation of a fibrous scar, therefore lead to epidural fibrosis (Slipman et al., 2002; Gambardella et al., 2005). Epidural fibrosis is commonly found in the epidural space, which causes pain in the waist and lower extremities (Li et al., 2014). Reducing the formation of fibrosis and scar could significantly improve the life quality of patients, thus it is of great significance to find novel strategies to screen and forecast the activation and proliferation of fibroblasts.

MicroRNAs (miRNAs) are small non-coding RNAs with 18-25 nucleotides in length, which have been demonstrated be widely expressed and relatively conserved in humans (Ludwig et al., 2016; Ban et al., 2017). miRNAs have no opening reading frames, which makes them unable to be translated into proteins (Bartel, 2004). However, miRNAs could negatively regulate the expression of target genes by binding to the 3’UTR of target mRNA molecules, thus miRNAs have been reported to be involved in various biological processes, such as carcinogenesis (Chen et al., 2019; Nariman-Saleh-Fam et al., 2019), anticancer effect (Tao et al., 2018; Yang J et al., 2020), neuro-protection (Song et al., 2019; Xiao et al. 2019), as well as epidural fibrosis (Lin et al., 2018). For example, an investigation on the role of miR-519d-3p in postoperative epidural scar formation and found that the overexpression of miR-519d-3p promoted the proliferation of fibroblasts and the expression of type I collagen, which are important factors in epidural fibrosis (Yang L *et al.,* 2019). Previously, it has been demonstrated that the prolapse of the intervertebral nucleus pulposus would lead to an epidural inflammation (McCarron et al., 1987). miR-146 was revealed to regulate the repair and regeneration of intervertebral nucleus pulposus cells and participate in the fibroblast activation and pathology of arthritis (Saferding et al., 2017; Yang R *et al.,* 2019). Therefore, miR-146 was speculated to play roles in epidural fibrosis of patients undergoing laminectomy. 

This study focused on the expression and function of miR-146 in epidural fibrosis, aiming to disclose the clinical significance and functional role of miR-146 during epidural fibrosis.

## Subjects and Methods

### Patients and sample collection

This study recruited 72 lumbar disc herniation patients who underwent the laminectomy at The Affiliated Hospital of Qingdao University from 2018-2019. In the recruited patients, 45 patients developed epidural fibrosis and 27 patients did not develop epidural fibrosis (control). They have never received any chemotherapy or radiotherapy and have signed written consent. Patients with other systemic diseases were excluded. The lumbar disc tissues were collected during the laminectomy and immediately frozen in liquid nitrogen and stored at -80 °C. This study has been approved by the Ethics Committee of The Affiliated Hospital of Qingdao University. 

### Cell culture 

Primary human epidural fibroblasts were isolated from collected epidural scar tissues using the enzymatic digestion method as previously reported (Shi et al., 2013; Sun et al., 2015). The isolated cells were incubated in DMEM medium with 10% FBS, 100 μg/mL streptomycin, and penicillin, then incubated at 37 °C with 5% CO_2_.

### Cell transfection

Cultured cells were seeded into 96-well plates and transfected with miR-146 mimic (5’-UGAGAACUGAAUUCCAUGGGUU-3’), miR-146 inhibitor (5’-AACCCAUGGAAUUCAGUUCUCA-3’), and negative controls (mimicNC (5’-UUGUACUACACAAAAGUACUG-3’) and inhibitor NC (5’-UCACAACCUCCUAGAAAGAGUAGA-3’)) to regulate the expression of miR-146. The transfection agents were synthesized by Invitrogen (Thermo Fisher Scientific, Inc.) and performed with lipofectamine® 3000 (Thermo Fisher Scientific, Inc.).

### RNA isolation and RT-qPCR

Total RNA was extracted from collected tissues and cultured cells with the TRIzol reagent (Invitrogen, Thermo Fisher Scientific, Inc.) and reverse transcribed to cDNA with the TaqMan microRNA Reverse Transcription kit (Thermo Fisher Scientific). PCR was conducted with SYBR Green Master MIX kit (Invitrogen, Thermo Fisher Scientific, Inc.) with U6 as the internal reference. The primer sequences were: 5’-TCCACCAAGAAGCTGAGCGAG-3’ (forward), 5’-GTCCAGCCCATGATGGTTCT-3’ (reverse). The relative expression level of miR-146 was calculated by the 2^-ΔΔCt^ method. The reaction conditions were as follows: 51 °C for 2 min, 96 °C for 10 min, 96 °C for 10 s, and 60 °C for 30 s, with a total of 40 cycles.

### Enzyme-linked immunosorbent assay (ELISA)

The cytokines TNF-α IL-1 beta, and IL-6 were analyzed with the ELISA kit (R&D Systems, Minneapolis, MN, USA) in accordance with the manufacturer’s instructions.

### MTT assay

After 48 h of cell transfection, cells were seeded into 96-well plates at a density of 5x10^4^ cells per well. After incubating for 0, 24, 48, 72, and 96 h, 0.5 mg/mL MTT reagent was added to each well and cultured for 4 h at 37 °C. Then, dimethylsulfoxide (DMSO) was added to each well on a shaker for 10 min. Finally, the absorbance at 490 nm of each well was recorded by a microplate reader.

### Statistical analysis

To evaluate the diagnostic value of miR-146 to discriminate patients with epidural fibrosis from patients without epidural fibrosis, the receiver operating characteristic (ROC) curve was plotted, and the area under the curve (AUC) was also calculated. Additionally, the association between miR-146 expression and the clinical features of patients was assessed by the χ^2^ test, and the correlation between miR-146 expression and cytokines was evaluated by the Pearson correlation analysis. All data were presented as mean value ± SD obtained from at least triplicate experiments. The difference was considered to be statistically significant when *P* < 0.05.

## Results

### miR-146 was upregulated in patients that developed epidural fibrosis and was associated with the clinical features of patients

**Figure 1:**
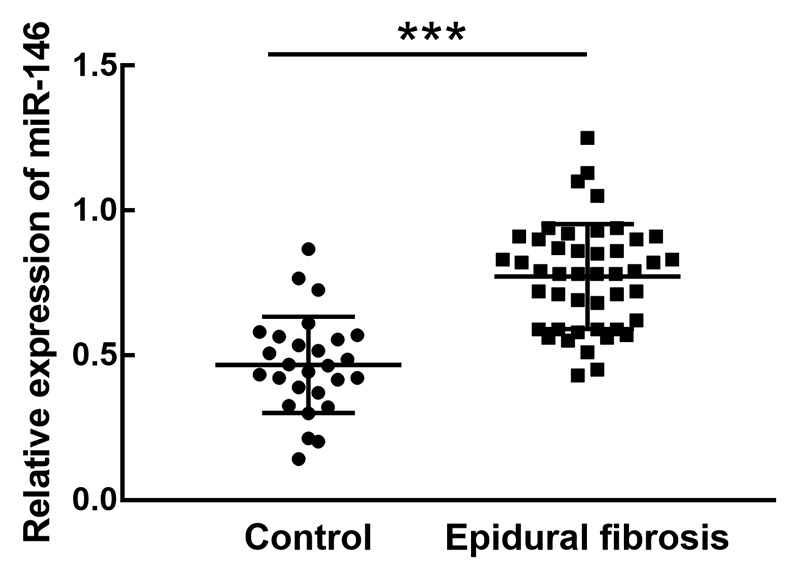
The expression of miR-146 in the lumbar disc tissues of patients with or without epidural fibrosis. miR-146 was significantly upregulated in epidural fibrosis patients compared with controls. ***P < 0.001.

In the collected lumbar disc tissues of patients with or without epidural fibrosis, the expression levels of miR-146 were investigated by RT-qPCR. It was found that miR-146 was significantly upregulated in patients that developed epidural fibrosis compared with the expression in the control group (*P* < 0.001, Figure 1). 

Further, based on the average expression level of miR-146 in the epidural fibrosis group (0.771), 45 patients were divided into a low miR-146 expression group (19 patients included 10 males and 9 females) and a high miR-146 expression group (26 patients included 17 males and 9 females). Among the clinical features of epidural fibrosis patients, the location (*P* = 0.017), low limb symptom (*P* = 0.007), and the duration of disease (*P* = 0.012) of patients showed significant association with the expression level of miR-146 (Table 1).

**Table 1:** Association between miR-146 expression and clinical features of patients with epidural fibrosis.

Parameters	Patients (n = 45)	miR-146 expression		P value
		Low (n = 19)	High (n = 26)	
Age				0.493
≤ 45	24	9	15	
>45	21	10	11	
Gender				0.388
Male	27	10	17	
Female	18	9	9	
Location				0.017*
L3-4	17	11	6	
L4-5	28	8	20	
Low back pain				0.121
No	20	11	9	
Yes	25	8	17	
Lower limb symptom				0.007**
No	18	12	6	
Yes	27	7	20	
Duration of disease (month)				0.012*
< 3	21	13	8	
>3	24	6	18	

### miR-146 could discriminate epidural fibrosis patients from control

**Figure 2:**
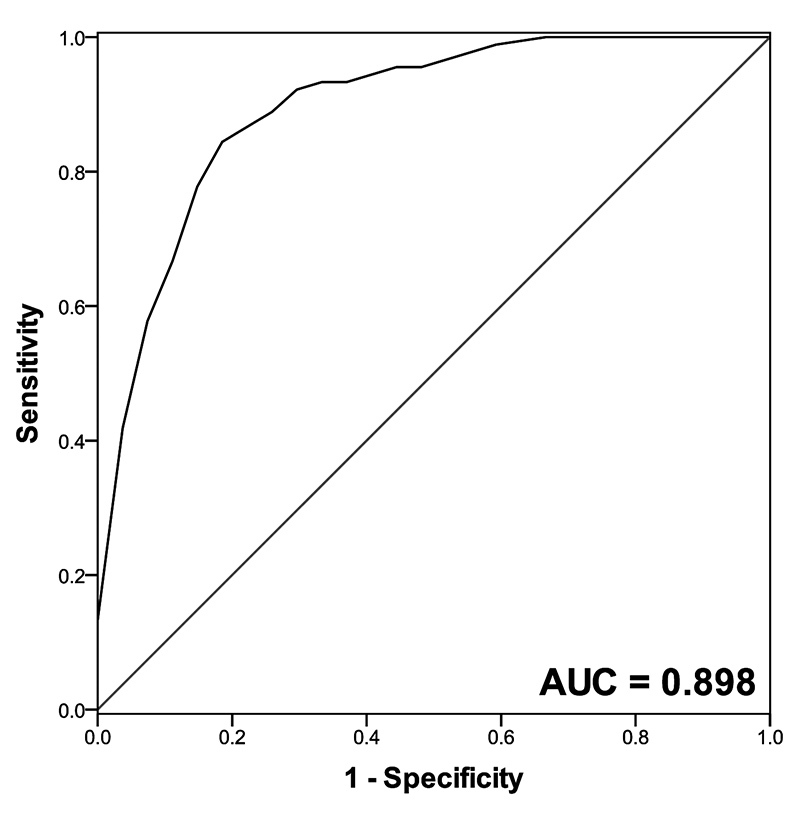
ROC curve based on the expression level of miR-146. miR-146 could discriminate epidural fibrosis patients from controls with the AUC value of 0.898. The sensitivity and specificity were 0.867 and 0.815, respectively.

From the ROC curve of epidural fibrosis patients, miR-146 showed a high sensitivity (0.867) and specificity (0.815) to distinguish epidural fibrosis patients from controls with the AUC of 0.898 (Figure 2).

### miR-146 was positively correlated with the pro-inflammatory cytokines in lumbar disc tissues

**Figure 3:**
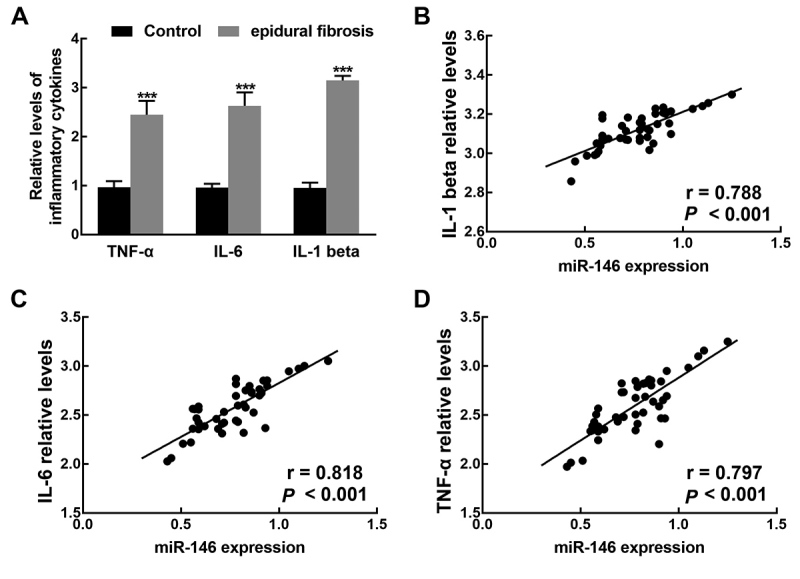
The relative levels of TNF-α, IL-6, and IL-1 beta and their correlation with the expression of miR-146. A. The relative levels of TNF-α, IL-6, and IL-1 beta were significantly higher in epidural fibrosis patients than that of control patients. ***P < 0.001.B. The expression of miR-146 was positively correlated with the relative levels of IL-1 beta with the *r* value of 0.788. C. The expression of miR-146 was positively correlated with the relative levels of IL-6 with the *r* value of 0.818. D. The expression of miR-146 was positively correlated with the relative levels of TNF-α with the *r* value of 0.797. All P < 0.001.

The relative levels of pro-inflammatory cytokines, including IL-1 beta, IL-6, and TNF-α, in the lumbar disc tissues of patients with or without epidural fibrosis, were evaluated by ELISA. As depicted in Figure 3A, the relative levels of IL-1 beta, IL-6, and TNF-α were significantly higher than those in controls (*P* < 0.001). Moreover, the levels of IL-1 beta (r = 0.788), IL-6 (r = 0.818), and TNF-α (r = 0.797) were found to be positively correlated with the expression level of miR-146 (all *P* < 0.001, Figure 3B-D).

### miR-146 promoted cell proliferation of primary human epidural fibroblasts and enhanced inflammatory response 

**Figure 4:**
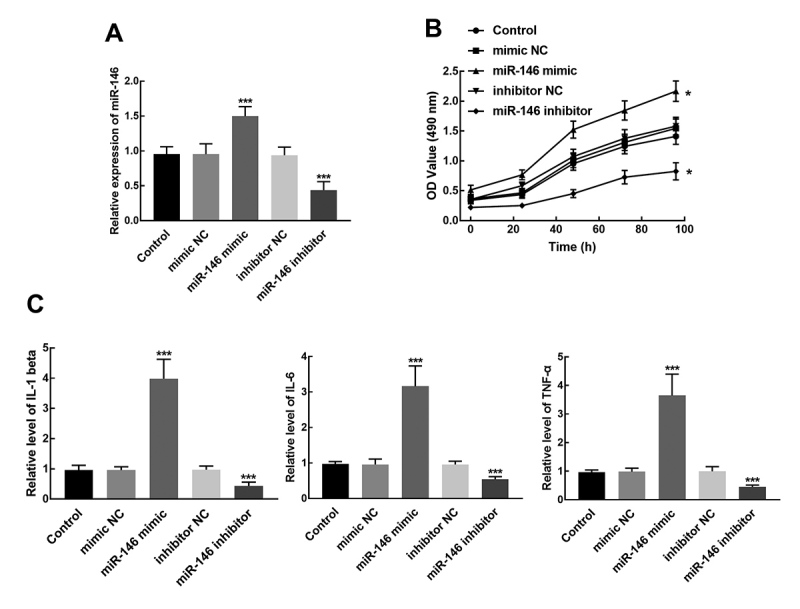
Effect of miR-146 on fibroblasts proliferation and relative levels of pro-inflammatory cytokines in isolated primary human epidural fibroblasts. A. The transfection of miR-146 mimic significantly increased the expression of miR-146, while the transfection of miR-146 inhibited the expression of miR146. ***P < 0.001 relative to control (untransfected cells). B. The proliferation of fibroblasts was promoted by the overexpression of miR-146 and suppressed by the knockdown of miR-146. *P < 0.05 relative control. C. The relative levels of IL-1 beta, IL-6, and TNF-α increased by the overexpression of miR-146 and reduced by the silencing of miR-146. ***P < 0.001 relative to control.

Isolated primary human epidural fibroblasts transfected with miR-146 mimic showed a notable overexpression of miR-146, while the transfection of miR-146 resulted in the silencing of miR-146 (*P* < 0.001, Figure 4A). The proliferation of epidural fibroblasts was dramatically enhanced by miR-146 overexpression and suppressed by the knockdown of miR-146 (*P* < 0.05, Figure 4B). Additionally, the relative levels of pro-inflammatory cytokines in transfected cells were also detected. It was found that the relative levels of IL-1 beta, IL-6, and TNF-α significantly increased in miR-146 mimic transfected cells and reduced in miR-146 inhibitor transfected cells (*P* < 0.001, Figure 4C).

## Discussion 

Postoperative epidural scar adhesion is the major cause of failed back surgery syndrome, which mainly resulted from epidural fibrosis (Xie et al., 2017). It has been reported that the abnormal proliferation and excessive activation of fibroblasts could induce epidural fibrosis, thus preventing or reducing the epidural scar is important to improve the postoperative recovery of patients. Previously, numerous miRNAs have been proved to be involved in the fibroblast activation or regulating fibrosis and collagen accumulation. For example, miR-125b could induce fibroblast proliferation and regulate the expression of fibrosis-related genes in the heart, which makes the inhibition of miR-125b a novel therapeutic approach for the treatment of human cardiac fibrosis and other fibrotic diseases (Nagpal et al., 2016). 

Saferding et al. (2017) identified miR-146 as an epigenetic regulator in arthritis that restricted the activation of fibroblasts and regulated the inflammatory response. Here, miR-146 in the lumbar disc tissues of lumbar disc herniation patients developed epidural fibrosis was significantly upregulated compared with patients that did not develop epidural fibrosis. The expression of miR-146 showed a close association with the clinical features of patients, including location, lower limb symptom, and the duration of disease. The dysregulation of miR-146 could differentiate patients developed epidural fibrosis from patients without epidural fibrosis, suggesting that miR-146 could serve as an indicator for the early detection of epidural fibrosis. Consistent with Saferding’s results, the miR-146 expression level was positively correlated with the relative levels of pro-inflammatory cytokines, such as IL-1 beta, IL-6, and TNF-α. Inflammatory response caused by the prolapse of the intervertebral nucleus pulposus would lead to epidural fibrosis (McCarron et al., 1987). Meanwhile, miR-146 was revealed to be associated with the repair and regeneration of intervertebral nucleus pulposus (Yang R et al., 2019). Thus, the positive correlation between miR-146 expression and the levels of the pro-inflammatory cytokine was speculated to indicate the involvement of miR-146 in the occurrence of epidural fibrosis. 

The increased proliferation of fibroblasts is also one of the main causes of epidural fibrosis. It has been shown that miR-203 overexpression led to a significant decrease of proliferation, invasion in keloid fibroblasts by suppressing EGR1 and FGF2, which implies the potential role of miR-203 in preventing and treating keloids (Shi et al., 2018). In this study, by means of cell transfection, the effect of miR-146 on the proliferation of epidural fibroblasts was assessed. The overexpression of miR-146 dramatically promoted the proliferation of epidural fibroblasts, whereas the knockdown of miR-146 showed an adverse effect on the proliferation. Additionally, the relative levels of pro-inflammatory cytokines also increased by the overexpression of miR-146 and suppressed by the silencing of miR-146, which is consistent with the results of the lumbar disc tissues. These results suggest that miR-146 might serve as a therapeutic target for epidural fibrosis.

However, the lack of mechanism experiments is one of the limitations of the present study. In previous mechanism studies it was reported that transforming growth factor (TGF)β signaling is a key factor in the regulation of fibrosis (Meng et al., 2016). miR-27 and miR-519d were confirmed to mediate fibroblast activation and epidural fibrosis by targeting the TGFβ signaling pathway (Zeng et al., 2017; Yang L et al., 2019). Besides, the accumulation of collagen was also a principal cause of epidural fibrosis. The expression of collagen I A1 is a key marker for the production of collagen in fibroblasts of hypertrophic scar, which was involved in the function of miR-382-5p in epidural fibrosis (Lin et al., 2018). miR-146 was also reported to regulate TGFβ signaling pathway in skeletal muscle after acute contusion and mediate the production of collagen in osteoarthritis cartilage (Yamasaki et al., 2009; Sun et al., 2017). Therefore, collagen I A1 and TGFβ signaling pathway were speculated to be the potential mechanism underlying the function of miR-146 in epidural fibrosis, which need further validations. 

Taken together, the upregulation of miR-146 acts as a diagnostic biomarker and promoted the inflammatory response and fibroblasts proliferation in epidural fibrosis. These results provide a novel molecular target for the detection and therapy of epidural fibrosis.
